# Ice-Binding Proteins in a Chrysophycean Snow Alga: Acquisition of an Essential Gene by Horizontal Gene Transfer

**DOI:** 10.3389/fmicb.2019.02697

**Published:** 2019-11-28

**Authors:** James A. Raymond, Daniel Remias

**Affiliations:** ^1^School of Life Sciences, University of Nevada Las Vegas, Las Vegas, NV, United States; ^2^School of Engineering, University of Applied Sciences Upper Austria, Wels, Austria

**Keywords:** snow algae, extremophiles, ice-binding proteins, horizontal gene transfer, gene regulation, essential genes

## Abstract

All ice-associated algae examined so far have genes for ice-binding proteins (IBPs), which suggest that these proteins are essential for survival in icy habitats. The most common type of IBP, type 1 IBPs (also referred to as DUF3494 IBPs), is also found in ice-associated bacteria and fungi. Previous studies have suggested that algal IBP genes were acquired by horizontal transfer from other microorganisms (probably bacteria). However, it remains unclear whether this is also the case for algae distantly related to the ones examined so far and whether microorganisms other than bacteria could be the donors. Furthermore, there is only limited evidence that these proteins are expressed at low temperature. Here, we show that *Kremastochrysopsis austriaca* (Chrysophyceae), an Austrian snow alga that is not closely related to any of the ice-associated algae examined so far, also produces IBPs, although their activity was weak. Sequencing the algal genome and the transcriptomes of cells grown at 1 and 15°C revealed three isoforms of a type 1 IBP. In agreement with their putative function, the three isoforms were strongly upregulated by one to two orders of magnitude at 1°C compared to 15°C. In a phylogenetic tree, the *K. austriaca* IBPs were distant from other algal IBPs, with the closest matches being bacterial proteins. These results suggest that the *K. austriaca* IBPs were derived from a gene that was acquired from a bacterium unrelated to other IBP donor bacteria and confirm by their presence in yet another alga the essential role of algal IBPs.

## Introduction

Ice-binding proteins (IBPs) are produced and secreted by many ice-associated microorganisms (bacteria, algae, and fungi) from both marine and freshwater environments (Davies, 2014; Bar Dolev et al., 2016; Guo et al., 2017; Raymond and Morgan-Kiss, 2017; Vance et al., 2019). Some authors refer to microorganismal IBPs as antifreeze proteins, but that is incorrect because, unlike fish and insect antifreeze proteins (Devries, 1983; Duman, 2015), microorganismal IBPs are produced at concentrations that are too low to appreciably lower the freezing point. Rather, they serve to mitigate damage from external ice by altering its structure and inhibiting its recrystallization (e.g., Raymond and Morgan-Kiss, 2013). Algal IBP genes appear to have been acquired from bacteria for several reasons: (1) the closest matches to algal IBPs (apart from other known algal and fungal IBP genes) are bacterial proteins, (2) IBP homologs are widely distributed in bacteria and have not been found in the genomes of mesophilic algae, and (3) the phylogeny of algal IBPs is largely independent of algal taxonomy (Raymond and Kim, 2012). Still, the origin of the algal IBPs remains unclear as no close matches between algal and bacterial IBPs have been found so far. The best matches are generally below 60% amino acid sequence identity (80% would be more convincing). It is thus of interest to identify more algal IBPs to bring in to better focus their relation with bacterial genes. As for where to look, we reasoned that identification of IBPs in previously unexplored algal taxa might make it easier to recognize patterns in their distribution.

Among unicellular, ice-associated algae, two types of IBP have been found so far. The most common type (type 1), which was first found in a snow mold (Hoshino et al., 2003) and subsequently in sea ice diatoms, bacteria, snow, and lake algae and yeast, contains a ~200 amino acid sequence called the DUF3494 domain (for a review of its role in ice binding, see Vance et al., 2019) and usually an N-terminal signal peptide that causes the protein to be secreted from the cells. Nine recombinant type 1 (DUF3494) IBPs have been crystallized and their structures determined (see “Discussion”). In all cases, the DUF3494 domain has the shape of a triangular beta solenoid in which one of the three sides is the principle ice-binding site. Proteins with a DUF3494 domain also occur in over 100 species of bacteria and archaea from all types of habitat (e.g., sea ice, glaciers, acid mine drainage, hot springs, and bogs), which suggest that they serve to bind substrates other than ice in warmer environments. A second type of algal IBP, type 2, has been found in a bipolar chlorophyte, probably *Chloromonas* sp., in which TXT motifs appear to be responsible for ice binding (Raymond et al., 2009; Jung et al., 2016; Cho et al., 2019).

Recently, a new species of snow alga, *Kremastochrysopsis austriaca*, was identified in the Austrian Alps (Remias et al., 2019). It is a chrysophyte (Stramenopiles), a class that has not been previously investigated with respect to IBPs. After finding evidence of IBP activity in a cell culture (pitting of the surface of an ice crystal growing in the culture medium), we sequenced the genome and transcriptomes of the alga incubated at 1 and 15°C to hunt for the IBP genes. Here, we show that *K. austriaca* has at least three isoforms of a type 1 (DUF3494) IBP that appear distant from other type 1 algal IBPs and that were most likely acquired from a bacterial gene. The new results, combined with previous results, also point to a possible difference between marine and freshwater IBPs.

## Methods

### Cells

The cells used in the present study were obtained from a melting Alpine snowfield in Tyrol province, Austria in May 2017. At the University of Applied Sciences Upper Austria, a unialgal although non-axenic strain (DR75b) was established at 5°C in DY-V medium and then simultaneously cultured at 1 and 15°C in aerated 250-ml Erlenmeyer flasks. The 15°C cells were cultured in a walk-in environmental chamber, and the 1°C cells were cultured in a Percival LT36VL culture chamber (CLF PlantClimatics, Wertingen, Germany) inside the environmental chamber. The cells were illuminated for 14 h per day with fluorescent lighting at a flux of approximately 40 μmol photons m^−2^ s^−1^. Small numbers of bacteria and another eukaryote, possibly an amoeba, were observed under the microscope. We estimated that they each accounted for less than 0.1% of the total biomass of the cultures.

### Sequencing

After allowing non-axenic cultures to adapt to 1 and 15°C for 2 weeks, RNA was extracted from intact cells with a NucleoSpin® RNA Plant kit (Macherey-Nagel, Duren, Germany). RNAs (1 and 15°C) having RNA integrity numbers (RIN) of 8.9 and 8.7, respectively, as measured with a model 2,100 Bioanalyzer (Agilent), were sent to Eurofins (Ebersberg, Germany) for sequencing. There, poly (A) RNA was isolated (removing most of ribosomal RNA) and used to prepare strand-specific cDNA libraries for paired-end sequencing. The libraries were sequenced on an Illumina HiSeq platform, yielding 93.2 million 1°C reads and 86.6 million 15°C reads, each with a length of 151 nt. At the University of Nevada Las Vegas (UNLV), the reads were assembled with CLC Genomics Workbench into contig files of 57.4 and 48.5 Mb, respectively. The 1 and 15°C transcriptomes were submitted to NCBI (Bioproject PRJNA557425).

To obtain genomic DNA, intact cells were incubated with hexadecyltrimethylammonium bromide (CTAB) extraction buffer and β-mercaptoethanol at 65°C for 1 h. Proteins and DNA were separated by adding an equal volume of phenol-chloroform-isoamyl alcohol (25:24:1). Nucleic acids were recovered by ice-cold isopropanol precipitation, washed twice with ethanol, and dried in a SpeedVac (Eppendorf™ Concentrator plus) prior to rehydration in nuclease-free water. The DNA was sent to Seqomics Biotechnologia (Morahalom, Hungary) for sequencing. A mate-paired library was generated using an Illumina Nextera Mate-Paired Kit with insert sizes ranging between 7 and 11 kb. The library was sequenced on an Illumina MiSeq instrument using V2 sequencing chemistry resulting in 38.4 million 250-nt reads (19.2 million clusters of 2 × 250 nt). Raw data were pre-processed for *de novo* assembly following the manufacturer’s instructions and then assembled with SPAdes^1^, yielding a 206 Mb contig file.

IBP DUF3494 domains were identified with NCBI’s conserved domain database^2^. Signal peptides were identified with SignalP 5.0^3^. Maximum likelihood phylogenetic trees were constructed with Mega 6 (Tamura et al., 2013)^4^.

### Ice-Binding Protein Gene Expressions

Gene expressions at 1 and 15°C were based on FPKM (fragments per kilo base per million mapped reads) values using the formula FPKM = *r*/*R*/*L*, where *r* is the number of reads matching IBP nucleotide sequences with *e* values <1e-20, *R* is the number of millions of reads in the 1 and 15°C transcriptomes (93.2 and 86.6 million, respectively), and *L* is the length of the gene (from start codon to stop codon) in kb. To obtain *r*, all three IBPs were used as queries, the combined hits were sorted by *e* value, duplicates were removed, and the hits were then sorted by IBP number. For comparison, the FPKM values of a reference gene, ribosomal protein L23 (rpl23; Kianianmomeni and Hallmann, 2013) were also calculated.

### Ice-Binding Protein Activity

A sample of *K. austriaca* cells from Austria was transported to and cultured at Mariposa, AZ, USA, by Dr. Robert Andersen. Cells were grown at room temperature in DY-V medium and shipped to UNLV, where they were gradually adapted to 2°C in M-1 medium (Hoham et al., 2006). After at least 7 days at 2°C, ice-binding activity was estimated by observing irregularities in the surface of a slowly growing ice seed crystal submerged in cell-free culture supernatant (Raymond and Fritsen, 2001). Seed crystals were perfect, plate-like crystals of approximately 5 mm diameter, in which the ice *c*-axis was normal to the flat surface. Because the activity of the culture medium was weak, the medium was concentrated 100 times by freeze drying and resuspending in water to increase activity. Similarly concentrated fresh medium was used as a control.

### 3D Structure Prediction

The structure of the DUF3494 domain of *K. austriaca* IBP1 was predicted with Swiss Model (Arnold et al., 2006)^5^. Among the type 1 IBPs whose 3D structures have been determined, the one closest to IBP1, and the one used for the template, is an IBP isolated from a bacterial consortium obtained from the Antarctic ciliate *Euplotes focardii* [41.05% amino acid identity (57.4% similarity) over the DU3494 domain; Swiss Model Template Library ID 6eio.1; Mangiagalli et al., 2018]. The pdb file generated by Swiss Model was then submitted to the Yasara minimization server^6^ (Krieger et al., 2009) to generate an energy-minimized model using the default parameters and viewed with Yasara v. 19.7.20.

## Results

### Species Identification

The alga was identified as *Kremastochrysopsis austriaca* (Stramenopiles; Chrysophyceae) (Remias et al., 2019) partly on the basis of partial nucleotide sequences of its 18S ribosomal and ribulose-1,5-bisphosphate carboxylase/oxygenase large subunit (rbcL) genes obtained by PCR (acc. nos. MK614366 and MK614367, respectively). In the present study, we determined the full sequences of these genes by assembling transcripts (acc. nos. MK940544 and MK949449, respectively). The resulting sequences were identical to those obtained by PCR in the regions of overlap. Identical full 18S and rbcL sequences (without introns) were obtained from the genome. As α-tubulin is also used for taxonomic purposes, the *K. austriaca* sequence was assembled and submitted to GenBank (acc. no. MN441508). The deduced amino acid sequence matches other stramenopile α-tubulins with *e* values of 00.

Other sequences in the transcriptomes and genome indicated the presence of contaminating bacteria and at least one eukaryote, possibly an amoeba. The DNA contig file included several large (>1 Mb) contigs whose genes suggested that they were bacterial. The largest contig (3.4 Mb) appeared to belong to a bacterium of the glacier-associated genus *Glaciimonas*. The large sizes of the contigs (indicating many reads) indicated that these bacteria, despite their low visibility under the microscope, were well represented in the culture.

### Ice-Binding Protein Activity

**Figure 1:**
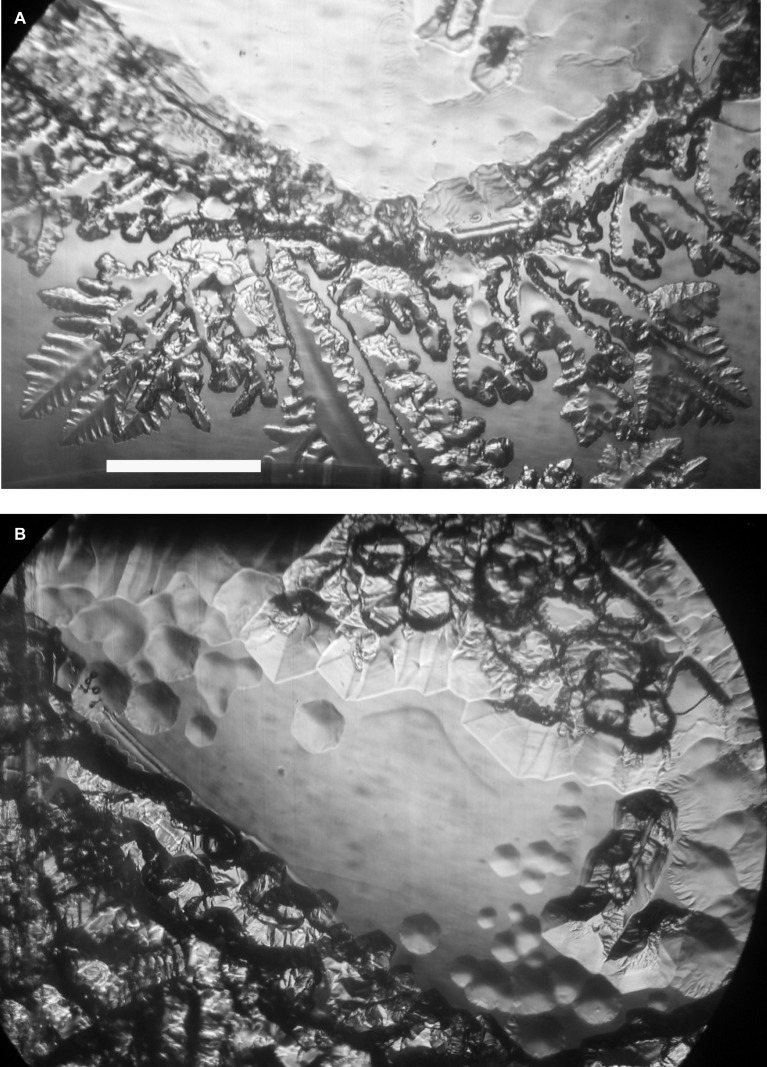
Ice-binding activity of concentrated *K. austriaca* culture medium. Activity is shown by pit formation and other distortions on a slowly growing ice crystal submerged in the medium after 2 h **(A)** and 3.5 h **(B)** at −1.0°C. The ice *c*-axis is normal to the page. Scale bar, 1 mm.

Ice-binding activity in the culture medium was difficult to detect without concentrating the medium. At 100× concentration, and after 2 h at −1°C, the freeze-dried, resuspended spent culture medium caused the appearance of nascent pits on the ice basal plane (Figure 1A, top) and deformed the growth of new ice on the prism faces (Figure 1B, bottom). After another 1.5 h, the pits were more developed and hexagonal, reflecting the hexagonal symmetry of the basal plane, and the growth on the prism faces was more extensive and more distorted (Figure 1B). By contrast, 100× concentrated fresh culture medium showed none of these features after 43 min, whereas the spent medium showed the first signs of activity after 15 min (data not shown). The distorted ice morphology shows that the cells secrete a substance into the medium that interferes with the growth of ice.

### Ice-Binding Protein Genes

**Figure 2:**
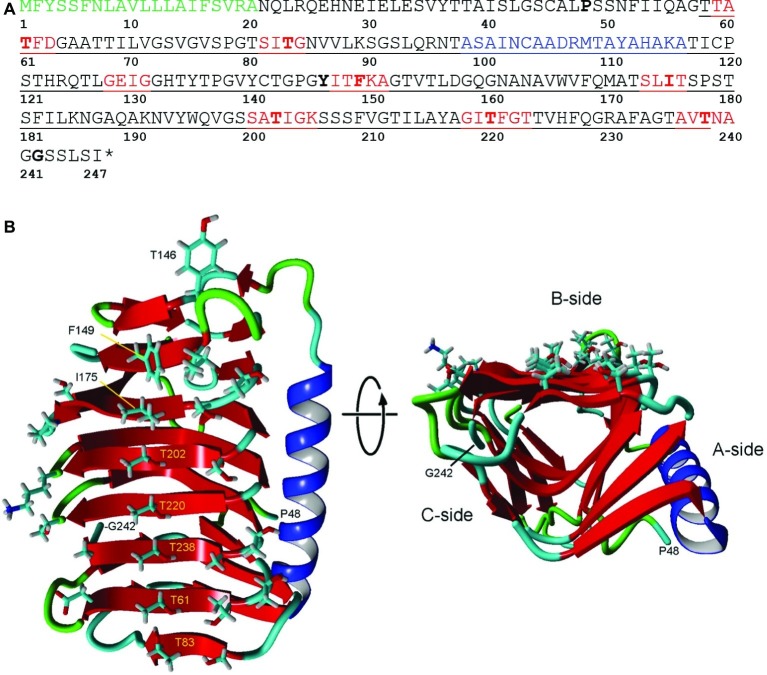
Structure of IBP1 of *K. austriaca*. **(A)** Deduced amino acid sequence. The protein has an N-terminal signal peptide (green) that is normally cleaved off as the protein is secreted from the cell and a DUF3494 ice-binding domain (underlined). Additional features predicted by Swiss Model/Yasara, see **(B)**, include beta sheets (red) that make up the ice-binding site and an α-helix (blue) that is characteristic of DUF3494 domains. Key residues that may be involved in ice binding, and the N- and C-terminal residues in the model are shown in bold. ^*^ stop codon. **(B)** Two views of the predicted structure of the DUF3494 domain based on the structure of *efc*IBP. This domain has the form of a triangular beta solenoid with sides A, B, and C, in which the B side is the predicted ice-binding site. Left, front view of the B side. Key residues in **(A)** are labeled. Right, view along the axis of the beta solenoid with the B side at top. Color coding is same as in **(A)**.

TBLASTN searches of the *K. austriaca* 1 and 15°C transcriptomes for known ice-binding proteins revealed the presence of genes for several very similar isoforms of type 1 (DUF3494) IBPs. No other genes associated with ice binding, such as genes for ice-nucleating proteins, were found. The 1°C transcriptome contained more than 32,000 unique reads matching the isoforms with *e* values <1e-20, making it unlikely that the reads were from a contaminating organism. In addition, one of the isoforms was on a DNA contig that included an intron-containing gene (acc. no. MK949445) that closely matched (*e* value = 00) a stramenopile chaperone gene. Three very similar sequences (IBP1, 2, and 3) were assembled using the transcripts, DNA reads, and sequencing of PCR products and submitted to GenBank (acc. nos. MK949447, MK949448, and MN441507, respectively). The deduced amino acid sequences of the three isoforms have lengths of 246 or 247 residues and have pair-wise identities between 91.9 and 95.2% (similarities between 95.6 and 97.2%). They have the typical sequence structure of type 1 (DUF3494) IBPs, with an N-terminal signal peptide and a 181-a.a. ice-binding domain (Figure 2A). The DNA and RNA sequences were the same, indicating that the genes have no introns. Additional reads that could not be assigned to any of the three isoforms suggested the presence of two or three additional isoforms. The similarity of the isoforms, together with the observation that the 5′ and 3′ untranslated regions (UTRs) were also very similar, strongly suggests that the isoforms were derived from a single gene by gene duplication.

In other cultures of IBP-producing algae, including the snow alga *Chloromonas brevispina* (JR unpublished data) and an Antarctic lake *Chlamydomonas* (Raymond and Morgan-Kiss, 2017), many type 1 IBPs belonging to contaminating bacteria were found. In the present case, however, no bacterial IBP genes were found, despite the presence of many bacteria in the culture.

### 18S and Ice-Binding Protein Phylogenies

**Figure 3:**
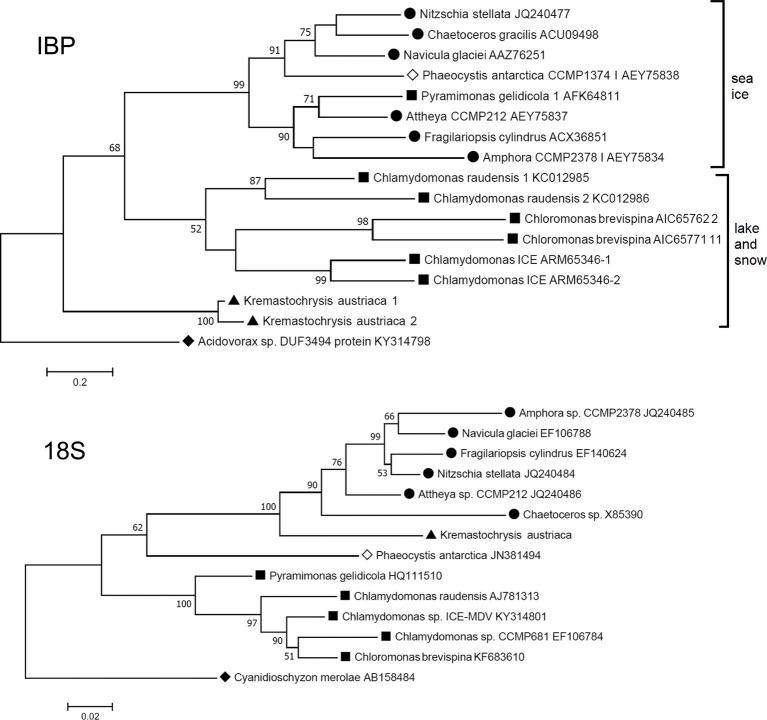
Comparison of maximum likelihood phylogenetic trees of the 18S ribosomal RNA and IBP sequences of IBP-producing algae. The IBP tree is based on amino acid sequences of the DUF3494 region. ● Diatoms (stramenopiles); ▲ *Kremastochrysopsis* (stramenopiles); ■ Chlorophyta; ◊ Haptophyceae; ♦ outgroups: *Cyanidioschyzon* (Rhodophyta) and *Acidovorax* sp. (Proteobacteria). Bootstrap values less than 50 are not shown.

The closest matches to the *K. austriaca* IBPs in the databases are bacterial proteins, but the matches are not very close (*e* values > 1e-32). Among algae, the closest matches were an IBP from an Antarctic lake alga of the genus *Chlamydomonas* (acc. no. ARM65346) (*e* values ~1e-22). Similar matches were obtained when just the DUF3494 domain was used as the query. This distance from other algal IBPs is reflected in a phylogenetic tree of all known type 1 algal IBPs, in which *K. austriaca* IBPs are well separated from the others (Figure 3 top). (The IBPs shown in the figure are representative of multiple homologous IBPs in each species).

Furthermore, the phylogeny of the algal IBPs, including those of *K. austriaca*, shows little resemblance to the phylogeny of the algae. For example, in a tree of the 18S rRNA sequences (Figure 3 bottom), *K. austriaca* groups with other stramenopiles as expected, but its IBPs (Figure 3 top) are unrelated to the IBPs of other stramenopiles, suggesting they were acquired independently. Similar incongruences occur in the locations of the IBPs of *Pyramimonas*, *Phaeocystis,* and *Chaetoceros*.

Many if not most bacterial IBPs have a signal peptide, which raises the question of whether the *K. austriaca* IBP signal peptide was acquired from a bacterium along with the DUF3494 domain. However, SignalP calculates that the *K. austriaca* IBP signal peptide has a high probability of being eukaryotic (0.9557) and lower probabilities of being Gram-negative or Gram-positive bacterial (0.8063 and 0.4533, respectively).

### mRNA Expression

**Figure 4:**
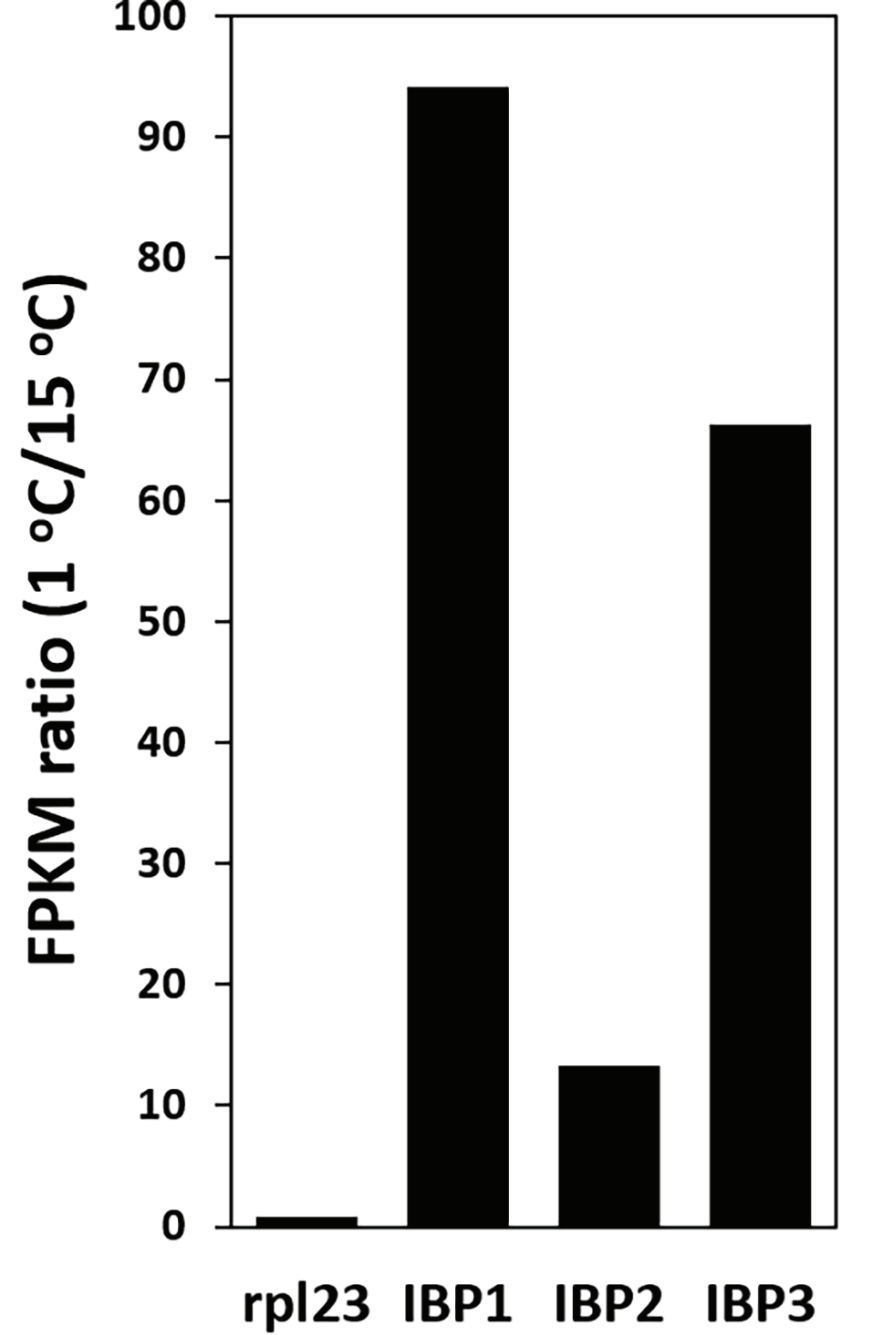
Upregulation of IBP transcripts at 1°C, as shown by the ratio of FPKM values at 1 and 15°C. Ribosomal protein L23 (rpl23) is a commonly used reference gene.

The abundance of IBP transcripts in the 1 and 15°C transcriptomes showed that the IBP1, 2, and 3 isoforms were strongly upregulated at 1°C: 94, 13, and 66 times, respectively (Figure 4). By contrast, the expression of a commonly used reference gene (rpl334) was little changed by temperature.

## Discussion

Remarkably, all ice-associated algae examined so far, including the present *Kremastochrysopsis austriaca*, isolated from melting alpine snow, have been found to produce ice-binding proteins, and in each case, in multiple isoforms. By contrast, no such genes have been found in the mesophilic algae whose genomes have been sequenced. This includes a temperate marine Chrysophycea alga closely related to *K. austriaca* (*Ochromonas* sp. CCMP1393) whose genome contains no IBP homologs. On the other hand, only a small percentage of ice-associated bacteria have such genes (Raymond et al., 2008). Together, these observations suggest that the IBPs are essential for the survival of algae in snow and ice.

### Ice-Binding Protein Function

Type 1 (DUF3494) IBPs have multiple effects on ice. Those with a signal peptide, which usually acts as a secretion signal, appear to modify the external environment in two ways, despite their very low concentrations. First, they inhibit the recrystallization (RI) of ice, whose moving grain boundaries are thought to damage cell walls (Knight et al., 1995). RI has been observed in several type 1 IBPs, including those from the sea ice diatoms *Navicula glaciei* (Raymond and Knight, 2003) and *Fragilariopsis cylindrus* (Bayer-Giraldi et al., 2011). Second, they can change the structure of ice, causing the formation of fine brine pockets that preserve a liquid environment for cells trapped in the ice. Microorganisms that secrete IBPs with this property include an Antarctic saline lake alga (Raymond and Morgan-Kiss, 2013) and a fungus (Arai et al., 2019). It has also been observed in unidentified exopolymeric substances secreted by sea ice diatoms (Krembs et al., 2011). Some IBPs lack a signal peptide and may have other functions, such as anchoring cells to an ice substrate or fortifying cell walls against ice. For example, a type 1 IBP gene from a snow mold (without its signal peptide), when expressed in body wall muscle of *C. elegans*, was found to dramatically increase the worm’s freezing tolerance (Kuramochi et al., 2019).

### Ice-Binding Protein Activity

Although we were unable to directly show that the type 1 IBPs of *K. austriaca* are the source of the ice-binding activity as shown in Figure 1, this is almost certainly the case, as purified or recombinant type 1 IBPs (Janech et al., 2006; Raymond et al., 2008; Lee et al., 2010; Bayer-Giraldi et al., 2011) and type 2 IBPs (Raymond et al., 2009) have been shown to be the source of similar ice-pitting activity. In addition, the transcript abundance of the IBPs dramatically increased at low temperature (Figure 4), and other known IBPs were not found in the *K. austriaca* genome or transcriptomes.

It is unclear why the IBP activity was weak, despite the high transcript abundance, as media from other cultures of IBP-producing algae (Janech et al., 2006; Raymond et al., 2009; Raymond and Morgan-Kiss, 2013, 2017; Raymond, 2014) showed activity without concentration. A possible explanation for the low activity is that translation of the transcripts was inhibited by the absence of some signal in the culture medium, such as ice (Jung et al., 2016). Another possibility is that the signal peptide serves as a membrane-targeting signal rather than a secretory signal, as the two types of signal are very similar. An external, membrane-bound IBP could thus serve to anchor the cell to an ice substrate, as has been reported for a non-DUF3494 bacterial IBP (Guo et al., 2017).

### Ice-Binding Protein Expression

The strong upregulation of IBP transcripts at 1°C is consistent with their having a role in mitigating freezing damage. The upregulation is comparable to that observed in the sea ice diatom *Fragilariopsis cylindrus*, which has a large family of type 1 IBPs. When *F. cylindrus* cells growing in marine medium (34 ppt) at 5°C were rapidly transferred to conditions found in sea ice brine pockets (70 ppt salinity at −4°C), transcript abundance of some IBP isoforms increased between 70 and 200 times after 20 days (although expression of some other isoforms greatly decreased) (Bayer-Giraldi et al., 2010). IBP protein expression also increased by the same treatment (Bayer-Giraldi et al., 2011). In addition, as stated above, transcript abundance of a type 2 IBP in an Antarctic alga (*Chloromonas*) was found to increase with increasing ice slush content in the medium (Jung et al., 2016). Eukaryotic type 1 (DUF3494) IBP transcripts were also found to be abundantly expressed in Arctic and Antarctic sea ice (Uhlig et al., 2015). On the other hand, temperature had no effect on transcript or protein expression of a type 1 IBP from a flavobacterium recovered from an Antarctic ice core (Achberger et al., 2011). This may be because the bacterium, unlike snow and sea ice algae, lived in a very stable environment and would have had no need to adapt to temperature or salinity changes.

### 3D Structure

DUF3494 domains have the shape of a triangular beta solenoid with sides A, B, and C, in which the B side has been identified as the principle ice-binding site (IBS). However, in some cases, the C side and several loop regions (including the α-helix on the A side) have been suggested to also be involved in ice binding (Vance et al., 2019). Of nine microorganismal DUF3494 structures that have been determined so far (for a list of studies, see Vance et al., 2019), the one with the closest match to *K. austriaca* IBP1 was an IBP isolated from an Antarctic marine bacterium (*efc*IBP; Mangiagalli et al., 2018). The predicted structure is shown in Figure 2B. On the lower end of the putative IBS (side B; Figure 2B left panel), the residues with outward-facing side chains include a central row of five threonine residues and a parallel row consisting of one threonine and three serine residues. The other end of the IBS includes three hydrophobic residues (Tyr146, Phe149, and Ile175). A search of the transcripts indicated that virtually all the isoforms have these residues. The hydrophilic residues are thought to bind water molecules that have the correct spacing to bind to an ice crystal lattice (Kondo et al., 2012). On the other hand, hydrophobic interactions can also increase the affinity of IBPs for ice (Nada and Furukawa, 2008; Cheng et al., 2016; Mochizuki and Molinero, 2018). Among the DUF3494 structures that have been determined, some have a capping region that blocks one end of the beta solenoid (the side corresponding to the front of the solenoid in Figure 2B, right panel), while others, such as *efc*IBP, do not (Vance et al., 2019). *K. austriaca* IBP1, like its model *efc*IBP, does not appear to have a capping region. Some DUF3494 structures have grooves along the IBS that are thought to be water molecule-binding sites (Kondo et al., 2012; Hanada et al., 2014; Cheng et al., 2016). Similar grooves can be seen in a stereo view of a space-filling model of the *K. austriaca* IBS (Supplementary Figure S1).

### Origin of Ice-Binding Proteins

Previous studies suggested that algal type 1 (DUF3494) IBP genes were acquired by horizontal gene transfer (HGT), most likely from bacteria (Bayer-Giraldi et al., 2010; Sorhannus, 2011; Raymond and Kim, 2012; Raymond and Morgan-Kiss, 2017). This also appears to be the case for the type 2 IBP (Raymond et al., 2009). The present results indicate an HGT origin of *K. austriaca*’s IBPs for the same reasons: they are unrelated to the IBPs of other stramenopiles (Figure 3), and their best matches are bacterial proteins. Because of the high similarity of the isoforms, it is likely that the IBPs were derived from a single-transfer event. Additionally, like bacterial genes, they lack introns. However, the closest bacterial proteins are still rather distant, leaving the source unknown. As more bacterial genomes become available, it is likely that better matches will be found.

IBPs are good candidates for gene transfer because they appear to be essential for survival in icy environments. The Antarctic fungus *Antarctomyces psychrotrophicus* was found to have two dissimilar DUF3494 type IBP genes that could best be explained by independent acquisition from bacteria (Arai et al., 2019). In the case of algae, all ice-associated algae examined so far have IBP genes, indeed, multiple isoforms of them. The strongest evidence of their horizontal transfer is the incongruence of the IBP and algal phylogenies (Figure 3), as phylogenetic incongruence is considered the gold standard for identifying HGT (Keeling and Palmer, 2008). The incongruence is most evident when the phylogenies being compared are both robust. Algal IBPs might provide the best example of incongruence reported so far in eukaryotes because the putative transferred genes are all homologs and can thus form a relatively robust tree. We are unaware of other such cases in eukaryotes, in which one group of organisms acquired virtually the same essential gene from a variety of different organisms. However, a similar case has been observed in bacteria living in a habitat, where naphthalene was the only carbon source and where a gene for a naphthalene-degrading enzyme was essential for survival. Many different species of *Pseudomonas* were found to have acquired different homologs of the enzyme from a variety of different donors (Ma et al., 2006). There, the incongruence of the *Pseudomonas* and enzyme phylogenies is best explained by HGT.

In the maximum likelihood IBP tree (Figure 3, top), which uses a distantly related bacterial type 1 IBP as an out group, the IBPs cluster into three groups with moderate to strong bootstrap values. Interestingly, all the algae in one group are associated with sea ice (and its brine pockets), while the algae in the other two groups are from snow and a frozen Antarctic lake. (The lake, Lake Bonney, has a strong halocline, but its ice cover is virtually freshwater ice without brine pockets.) The reason for the separation is unclear, but one possibility is that the two groups may reflect a difference in function, in which sea ice IBPs may be better designed to modify the structure of sea ice (to preserve brine pockets), while snow and lake IBPs may be better at inhibiting recrystallization of ice.

## Data Availability Statement

The datasets generated for this study can be found in NCBI (Bioproject PRJNA557425).

## Author Contributions

JR and DR contributed equally to this work.

### Conflict of Interest

The authors declare that the research was conducted in the absence of any commercial or financial relationships that could be construed as a potential conflict of interest.

## Footnotes

^1^http://cab.spbu.ru/software/spades/

^2^https://www.ncbi.nlm.nih.gov/Structure/cdd/wrpsb.cgi

^3^http://www.cbs.dtu.dk/services/SignalP/

^4^https://www.megasoftware.net/

^5^http://swissmodel.expasy.org

^6^http://www.yasara.org/minimizationserver.htm
