# Supplementary material for: Ice-Binding Proteins in a Chrysophycean Snow Alga: Acquisition of an Essential Gene by Horizontal Gene Transfer
Source: Front Microbiol. 2019 Nov 28;10:2697. doi: 10.3389/fmicb.2019.02697 (PMC6892780; doi:10.3389/fmicb.2019.02697)
Supplement: Supplementary file 1 [file Image_1.pdf]

Ice-binding proteins in a Chrysophycean snow alga:

acquisition of an essential gene by HGT

Supplementary information

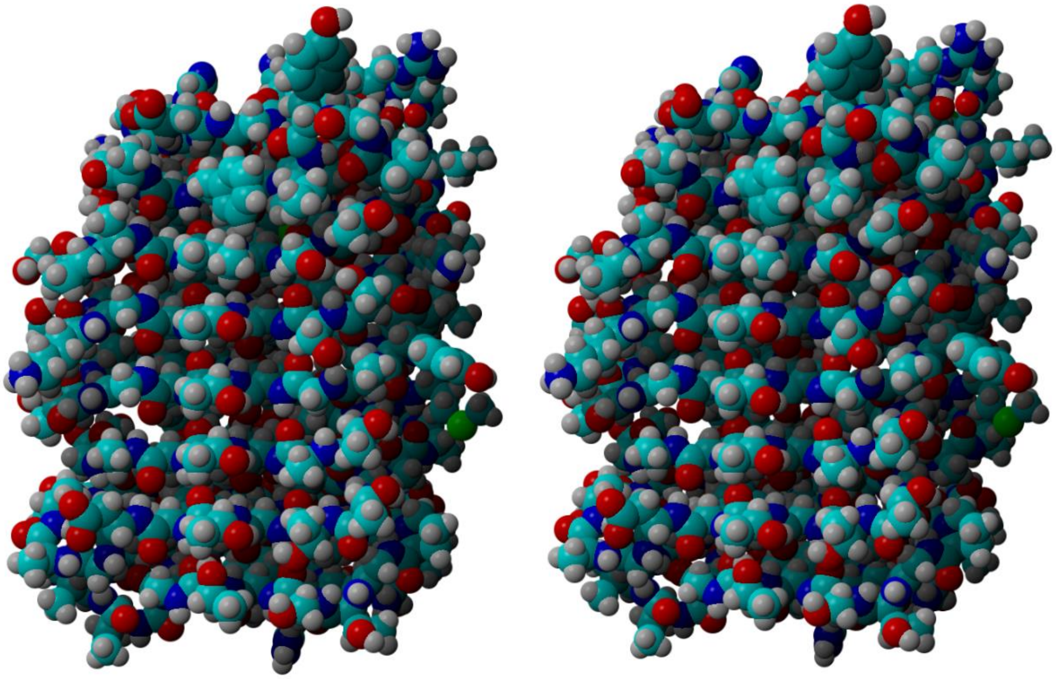

Fig. S1. Stereoview of space-filling model of putative ice-binding side (IBS) of *K. austriaca* IBP1, modeled after *efc*IBP. A vertical row of threonine residues appears at the center of the IBS, flanked by two grooves that other studies have speculated to be binding sites of water molecules. Hydrophobic Phe, Ile and Tyr residues are visible at the top. Images generated by Yasara viewer.
